# [Expression of Concern] Elevated microRNA-125b levels predict a worse prognosis in HER2-positive breast cancer patients 

**DOI:** 10.3892/ol.2026.15765

**Published:** 2026-07-15

**Authors:** Yanwei Luo, Xinye Wang, Weihong Niu, Heran Wang, Qiuyuan Wen, Songqing Fan, Ran Zhao, Zheng Li, Wei Xiong, Shuping Peng, Zhaoyang Zeng, Xiaoling Li, Guiyuan Li, Ming Tan, Ming Zhou

Oncol Lett 13: 867–874, 2017; DOI: 10.3892/ol.2016.5482

Following the publication of the above paper, it was drawn to the Editor's attention by a concerned reader that, regarding the histological images shown in Fig. 2 on p. 870, panels 2a and 2b contained an overlapping section, such that data which were intended to show the results from differently performed experiments had apparently been derived from the same original source. Furthermore, upon performing an independent analysis of the data in this paper in the Editorial Office, it came to light that certain the data in Fig. 1A were strikingly similar to data which subsequently appeared in a paper written by different authors at different research institutes in the journal *Molecular Medicine Reports*, which has since been retracted from that publication.

The authors have been contacted by the Editorial Office to offer an explanation for these apparent anomalies in the presentation of the data in this paper. Owing to the fact that the Editorial Office has been made aware of potential issues surrounding the scientific integrity of this paper, we are issuing an Expression of Concern to notify readers of this potential problem while the Editorial Office continues to investigate this matter further.

